# Evolution of pore structure and fractal characteristics of marine shale during electromagnetic radiation

**DOI:** 10.1371/journal.pone.0239662

**Published:** 2020-10-01

**Authors:** Xinhui Xie, Lanxiao Hu, Hucheng Deng, Jinjian Gao

**Affiliations:** 1 State Key Laboratory of Shale Oil and Gas Enrichment Mechanisms and Effective Development, Sinopec Petroleum Exploration and Production Research Institute, Beijing, China; 2 Shaanxi Key Laboratory of Advanced Stimulation Technology for Oil & Gas Reservoirs, Xi’an Shiyou University, Xi’an, Shaanxi, China; 3 College of Energy Resources, Chengdu University of Technology, Chengdu, Sichuan, China; 4 Department of Construction Engineering Management, Jiangxi Institute of Economic Administrators, Nanchang, Jiangxi, China; China University of Mining and Technology, CHINA

## Abstract

Electromagnetic radiation has been proposed to non-aqueously stimulate shale formations, which can generate fractures and enhance the porosity of the matrix. The proposed method consumes electricity and thereby possesses significant advantages for sustainable and environmental hydrocarbon production. In this study, we investigate the pore structure variations of marine shale during electromagnetic radiation. First, the prepared marine shale samples are exposed to electromagnetic radiation for different times; an infrared thermometer monitors the temperatures. Then, the nitrogen adsorption/desorption technique is applied to examine the evolutions of the pore structure. Next, a scanning electron microscope is adopted to reveal the morphology and identify newly developed pores. Lastly, fractal analyses are performed to quantify pore structure variations. The sample exhibits quick temperature rises, whose temperature reaches about 300 °C after 5 min of electromagnetic radiation. The elevated temperature causes clay dehydration, thermal expansion, and organic matter decomposition, leading to significant changes in pore structures. The nitrogen adsorption/desorption characteristics demonstrate enhancements in pore spaces, including volume, size, and surface area. Fractal analyses show that the pores become rougher and exhibit less heterogeneity after electromagnetic radiation. The obtained results demonstrate a great potential of using electromagnetic radiation to enhance the porosity of shale rocks.

## 1. Introduction

Shale gas, shale oil, and oil shale possessing considerable reserves have been crucial sequential energy resources for the increasing energy demand [1, 2]. Because of the low permeability of shale formations, stimulations, such as hydraulic fracturing, gas (CO_2_/N_2_) fracturing, and foam fracturing, are required to create high permeable fractures to enhance the hydrocarbon production [3, 4]. These stimulation techniques rely on injecting a substantial amount of fluids to fracture the shale rocks, which consume a massive amount of water and cause ecology problems [5]. Meanwhile, the injected fluid and the residual chemicals can block the pores, causing formation damages and reducing the permeability of the matrix [6]. Also, hydraulic fracturing requires extensive surface facilities and injects a massive amount of proppant and fracturing fluid. Electromagnetic radiation can be achieved by installing a downhole antenna to fracture the formation rock, which eliminates the injecting process and saves space for the surface facilities.

Formation heat treatment (FHT) has been proposed to stimulate formation rocks; previous investigations summarized the mechanisms as dehydration to enhance the porosity and thermal stress to create fractures [7–11]. Electromagnetic radiation has been proposed as a non-aqueous method to stimulate shale formations [12]. Unlike the conventional methods, electromagnetic radiation fractures the formation rocks by the internal thermal stresses generated by the heterogeneous expansions of minerals [13]. Electromagnetic radiation can generate fractures in the rock and enlarge the pores in the matrix, possessing significant advantages compared with the stimulating techniques that fracture the shales but damage the rock matrix. Hu et al. [14] investigated the petrophysical property changes of continental shale rocks under electromagnetic heating; results show that many fractures are generated by electromagnetic heating. Zhu et al. [15] showed that a higher power could significantly raise the heating rate and alter the pore structure of the shale sample. Li et al. [16] evaluated the fractal characteristics of pore structure of coal during microwave radiation and found a positive relationship between fractal dimension and thermal heterogeneity. Previous studies demonstrated great potentials of using electromagnetic radiation to fracture the shale rocks, and extensive fractures can be thermally induced. However, the pore structure changes of the matrix for marine shale under electromagnetic radiation are not fully understood.

In this study, we conduct experiments to understand the evolution of the pore structure of marine shale formation under electromagnetic radiation. The electromagnetic radiation experiments are performed by using a commercial microwave oven, while the temperature responses of the sample are monitored by the infrared thermometer. First, marine shale rocks are subjected to electromagnetic radiation at different times. Then, the isothermals of each sample are determined by the nitrogen adsorption/desorption technique. Also, the evolution of the pore structure and porosity of the samples is evaluated. Lastly, the fractal analysis is performed to reveal the geometric and morphology features of the samples.

## 2. Experimental section

### 2.1 Materials

**Fig 1:**
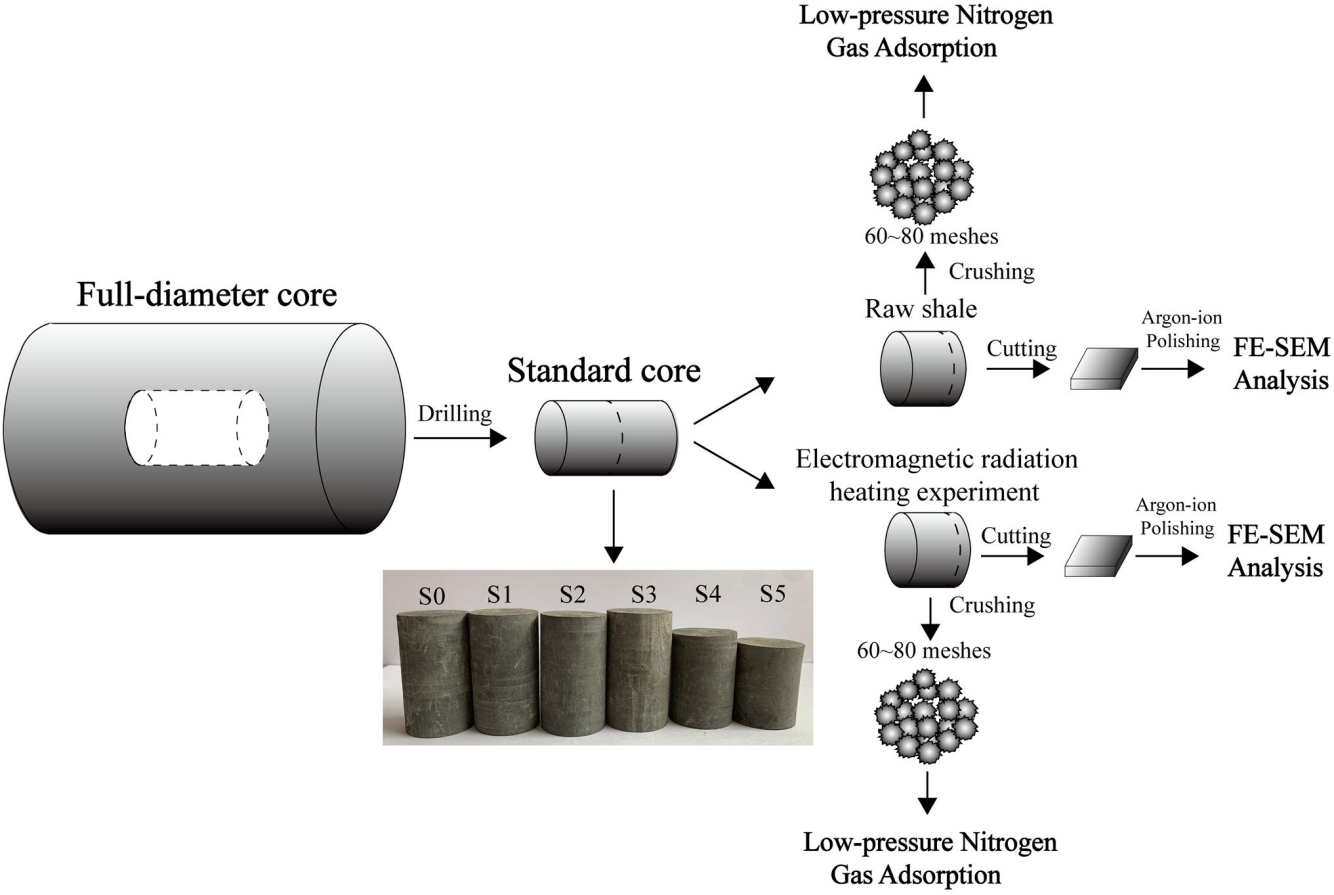
The workflow of the experimental study.

A downhole full diameter marine shale core, retrieved from Longmaxi Formation of Sichuan Basin, is utilized to investigate the pore structure evolution under electromagnetic radiation. Fig 1 summarizes the experimental workflow. We first drill multiple standard cores with a diameter of 2.54 cm and varying lengths from 3cm to 5 cm. Then, the geochemical properties of the core samples are characterized by the X-ray diffraction technique; the ones with similar composition are used to reduce the effect of heterogeneity on the experiments. Lastly, we conduct the low-pressure nitrogen gas adsorption and FE-SEM analysis to reveal the pore structure changes by electromagnetic radiation.

Table 1 lists the compositions of the selected samples as well as calculated average values and standard deviations. Results show that samples (S0-S7) consist of quartz, feldspar, calcite, dolomite, clay mineral, and organic matters. The mineral compositions of the selected samples are within a narrow range, with standard deviations smaller than 3.62.

**Table 1:** Mineral compositions and organic content of the marine shale samples used in this study.

Sample	Mineral composition (wt%)						TOC (wt%)
	Quartz	Feldspar	Calcite	Dolomite	Pyrite	Clay	
Sample #0	23	16	2	6	13	40	2.1
Sample #1	20	17	5	2	10	46	1.9
Sample #2	22	16	1	5	11	45	3.2
Sample #3	16	18	3	6	10	47	1.7
Sample #4	22	18	5	6	11	38	2.1
Sample #5	19	22	2	3	9	45	2.2
Sample #6	19	16	2	5	11	47	-
Sample #7	20	19	8	5	8	40	-
Average	20.2	17.8	3.5	4.7	10.3	43.5	2.2
Standard deviation	2.1	1.9	2.4	1.4	1.5	3.4	0.5

### 2.2 Experimental setup and procedures

**Fig 2:**
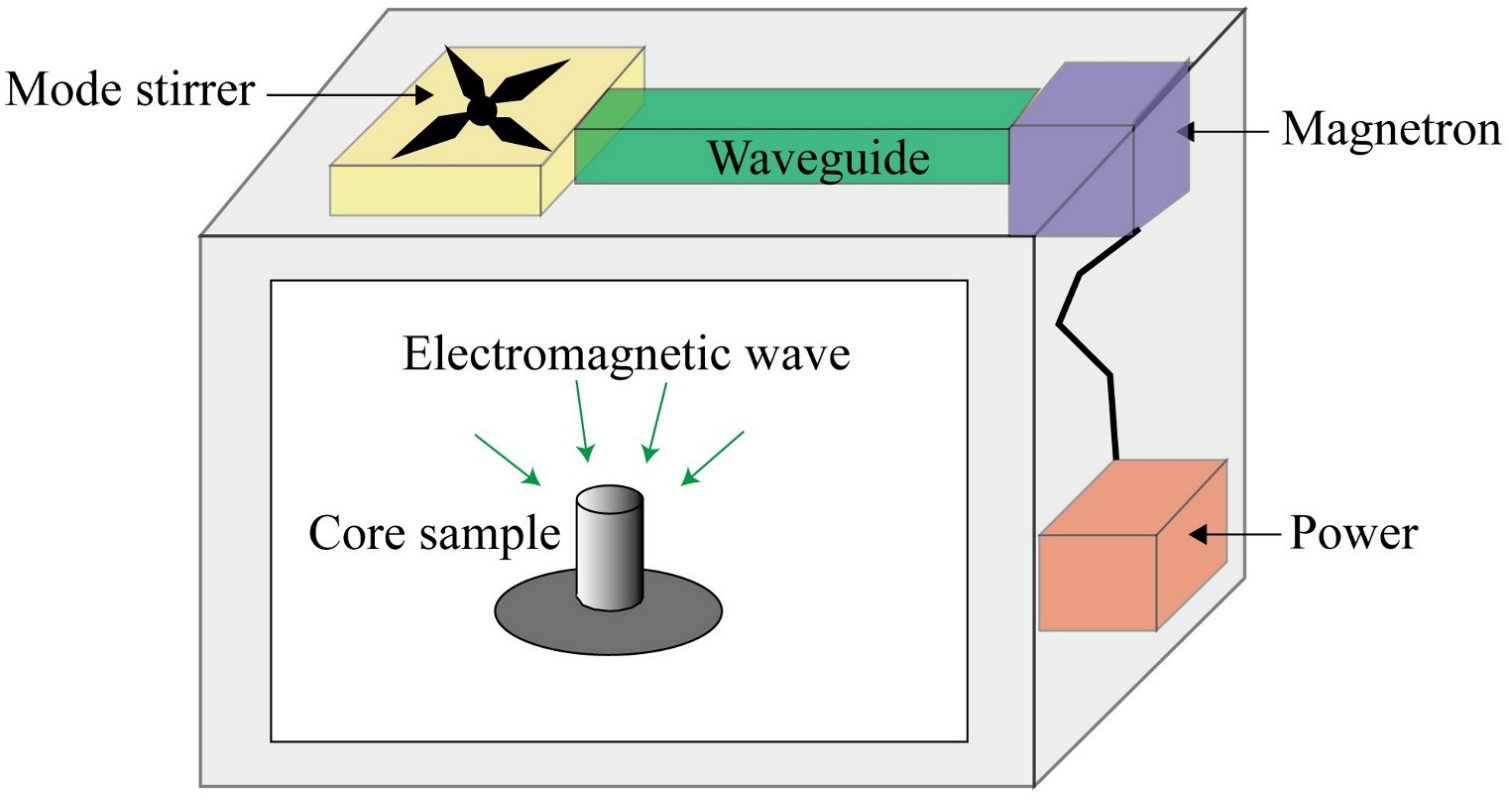
Schematic of the microwave oven used for investigating the pore structure of samples under electromagnetic radiation.

Fig 2 shows the schematic of the microwave oven used to conduct electromagnetic radiation experiments. A magnetron is used to generate the electromagnetic flux, and marine shale samples are placed in the center of the oven cavity. Before the experiments, all the samples are dried at 105°C for a day to eliminate the residual water. Then, the samples #1–5 are subjected to electromagnetic radiation for 1min, 2min, 3min, 4min, 5min, respectively; an infrared thermometer measures the surface temperature of shale samples. Lastly, samples #6–7 are used to check the reproducibility of the experiments.

**Fig 3:**
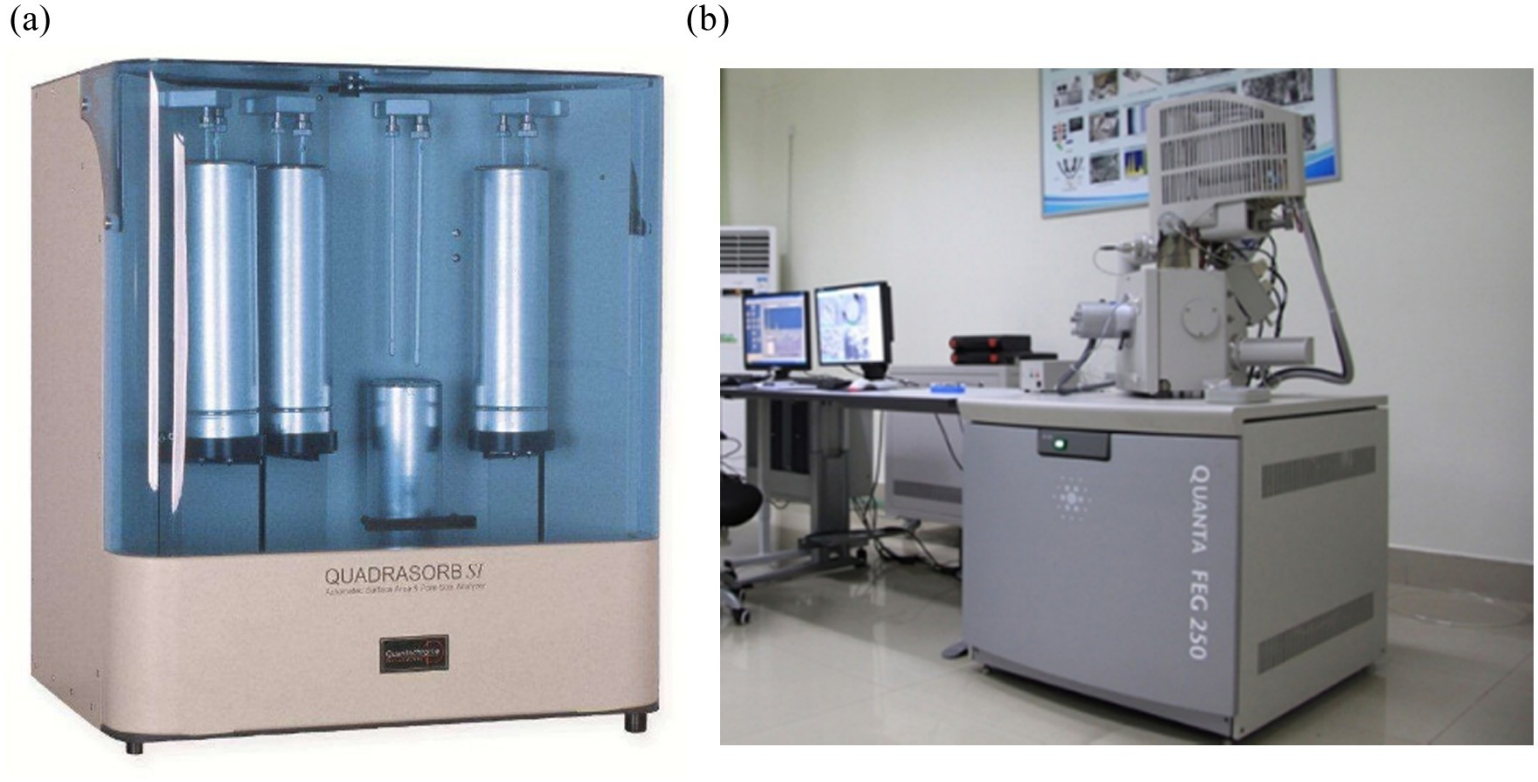
Experimental apparatus for (a) low-pressure nitrogen adsorption and (b) FE-SEM characterization.

After the electromagnetic radiation experiments, these samples are crushed and then sieved between 60 and 80 meshes for the nitrogen adsorption/desorption characterization. A Quadrasorb SI apparatus (USA) is adopted to reveal the pore structure characteristics, including the shape, size distribution, specific surface area, and volume of pores. Also, the effect of electromagnetic radiation on the pore morphology is examined by field emission scanning electron microscope (FE-SEM) (Quanta 250 FEG, USA). Fig 3 shows the images of the adopted characterization setups.

## 3. Experimental results

### 3.1 Temperature responses

**Fig 4:**
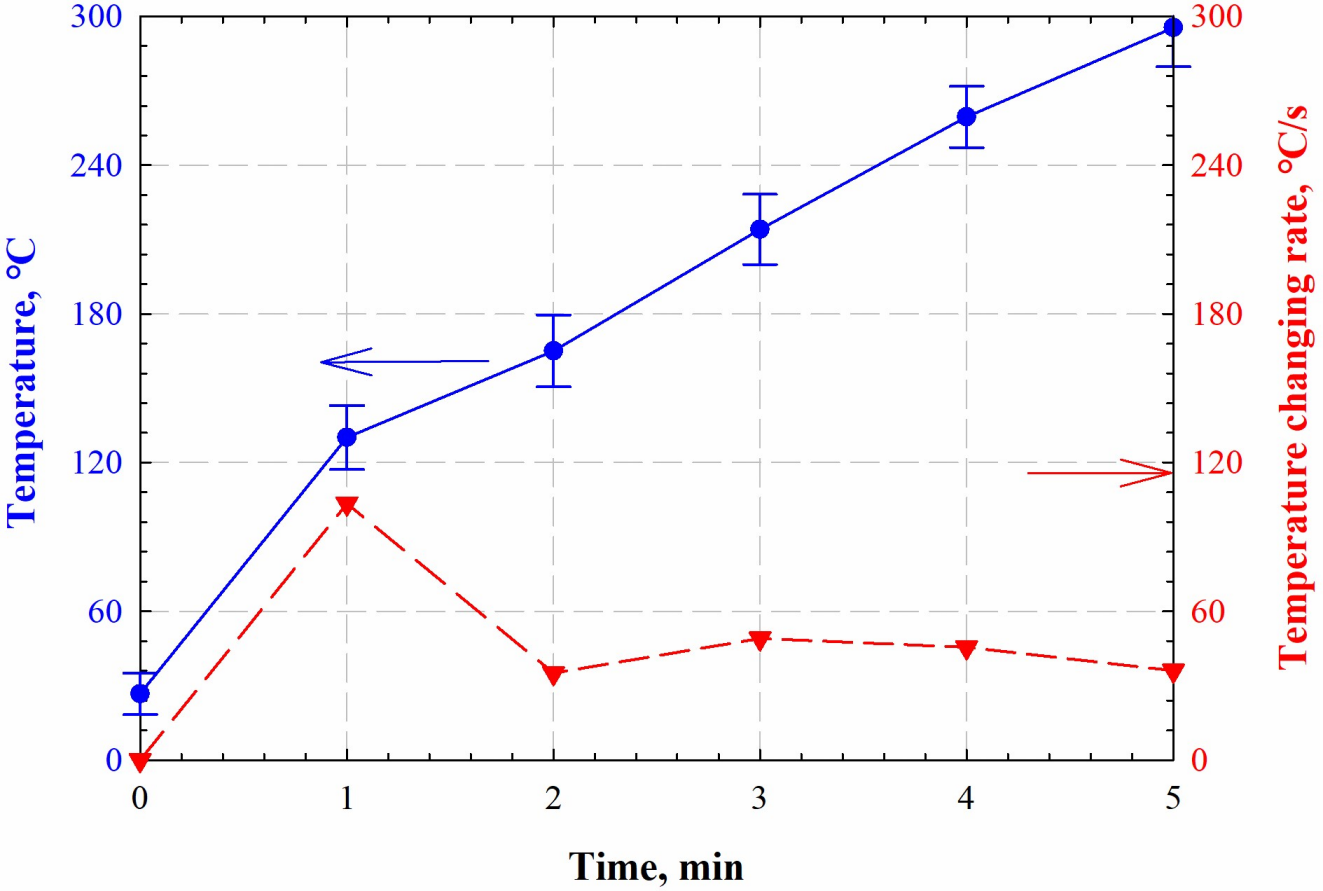
Temperature profiles and temperature-changing rates of shale samples at different electromagnetic radiation times.

Due to the heterogeneity of mineral distribution of the sample, temperature responses under electromagnetic radiation vary at different measuring points. Hence, we measure surface temperatures of shale samples for three times to obtain average values as the radiation temperature responses. Fig 4 shows the average temperature profiles and temperature-changing rates of shale samples under electromagnetic radiation. The temperatures increase as the electromagnetic radiation continues. The roaring temperature at the beginning of the experiment is caused by the clay-bound water that is an excellent electromagnetic wave absorber. Once the clay-bound water vaporizes, the temperature steadily increases and reaches about 300 °C after 5 min of electromagnetic radiation.

### 3.2 Nitrogen adsorption/desorption isotherms

**Fig 5:**
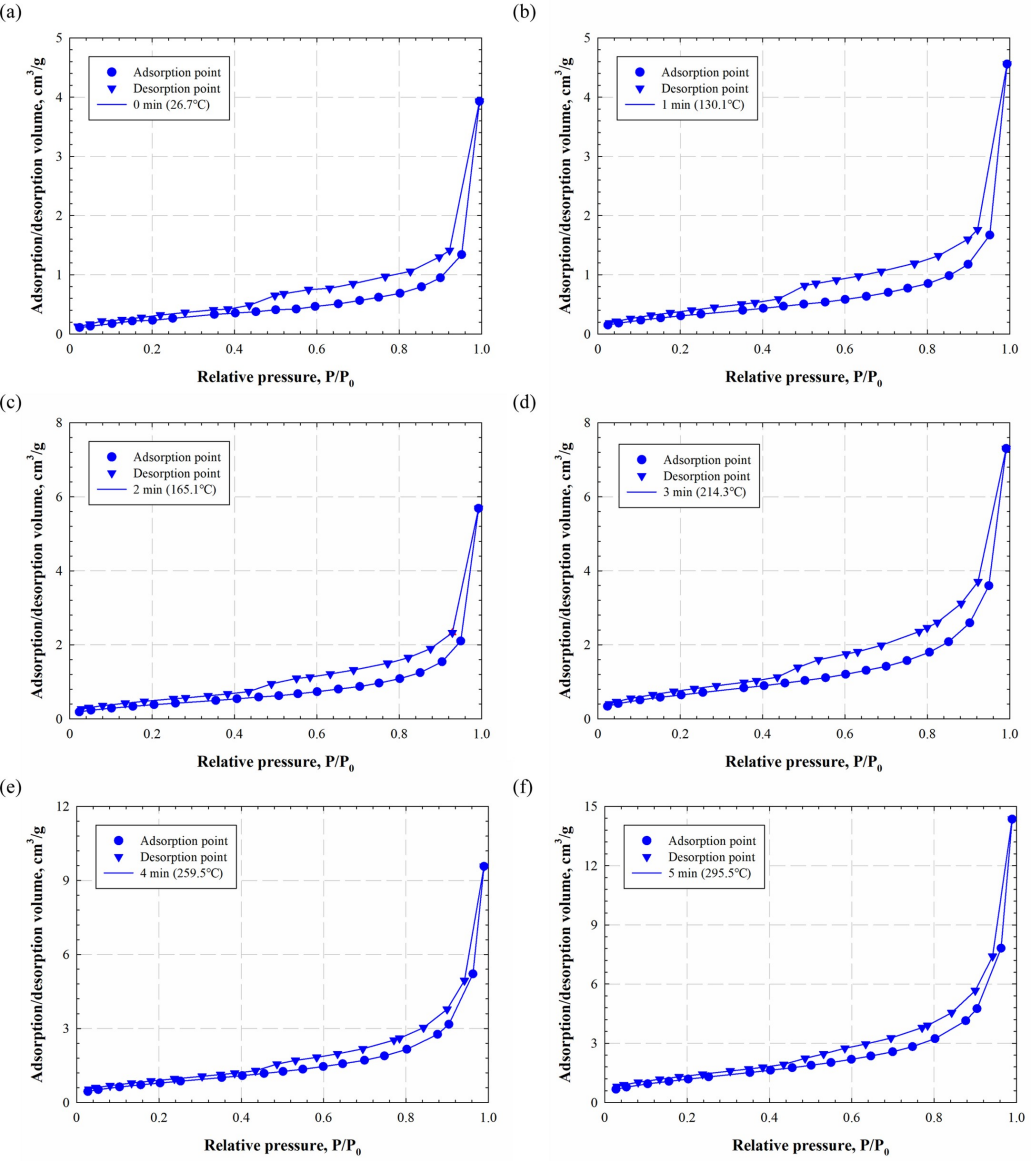
The adsorption/desorption isotherms of shale samples at different electromagnetic radiation times.

The nitrogen adsorption/desorption technique is adopted to investigate the evolution of pore structures under electromagnetic radiation. Fig 5 summarizes the obtained isotherms of shale samples at different radiation times. The adsorption isotherms steadily increase when the relative pressures (*P*/*P*_0_) are lower than 0.9, while the adsorption isotherms sharply rise once the *P*/*P*_0_ is over 0.9 due to the effect of capillary condensation. Also, the adsorption and desorption curves only exhibit insignificant differences when the *P*/*P*_0_ is smaller than 0.45, suggesting that small pores are connected by one pore throat [17].

The adsorption/desorption volumes continuously increase as the electromagnetic radiation goes on; the maximum absorption volumes increase from 4 to 14 cm^3^/g after 5 min of electromagnetic heating, indicating many new pores are generated. Also, the shape of the isothermal curves changes after electromagnetic radiation. Based on the classification of the International Union of Pure and Applied Chemistry (IUPAC) [18], the patterns of the isothermals can be used to detect the shape of the pore. The patterns of adsorption and desorption curves remain the same for the first 3 min of electromagnetic radiation; the obtained isotherms belong to type H_4_ that indicates a slit-like shape of pores. However, the pattern of adsorption and desorption isotherms change after 4 min of electromagnetic radiation. The developed new pores after electromagnetic radiation turn the pattern to H_2_ type that suggests an ink-bottle shape or flat-shape pore.

### 3.3 Validation

**Fig 6:**
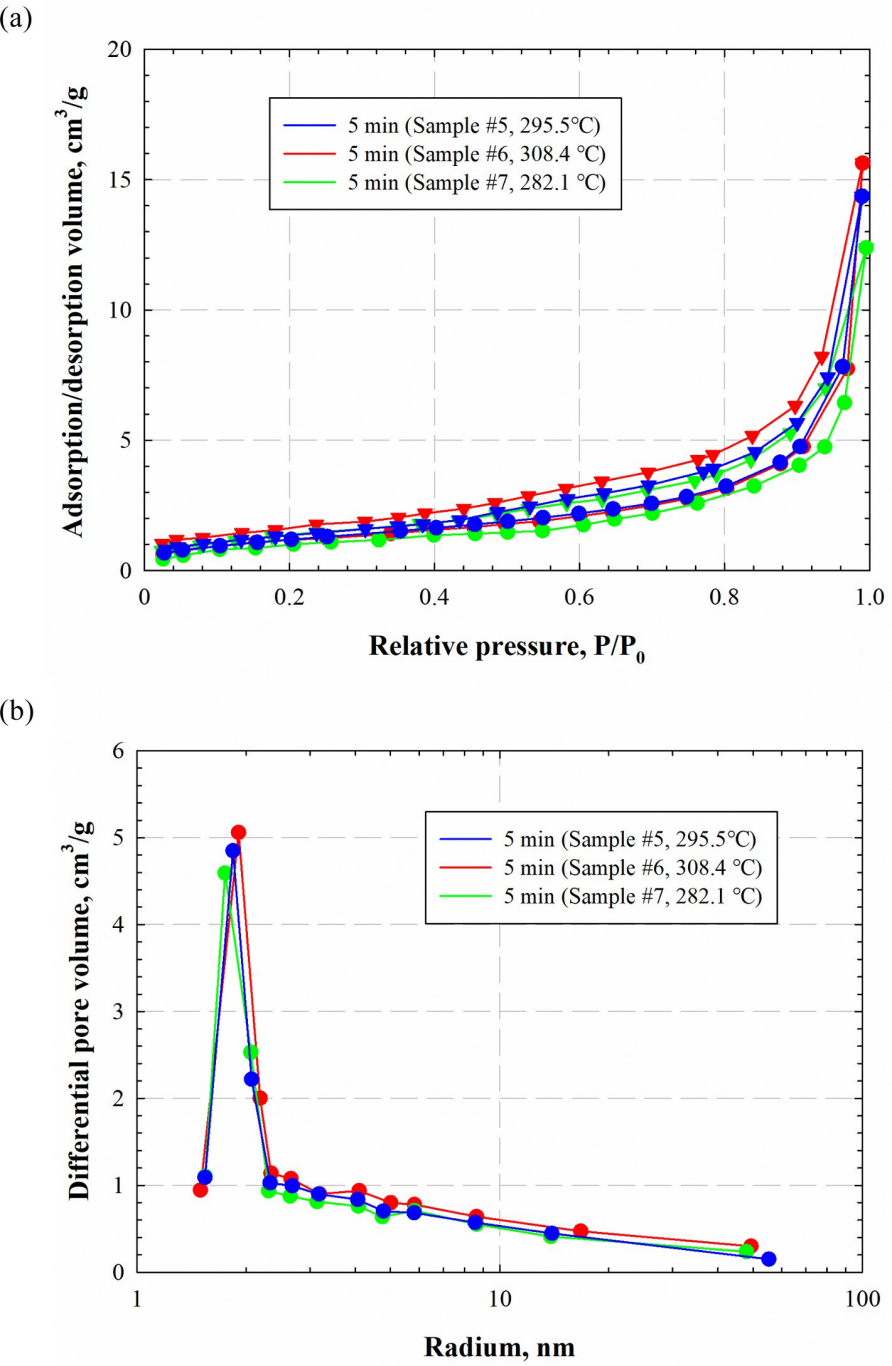
Comparison of the isothermals and pore-size distribution of samples #5–7.

To validate the experiments, we conduct another two runs (samples #6–7) to repeat the case of 5min of electromagnetic radiation (sample #5). Fig 6 shows the obtained isothermals and the pore-size distributions of samples #5–7. Results show that the temperature responses, isothermals, and pore-size distributions of three cases vary within a small range. To quantify the differences, we calculate the average absolute relative deviations ($AARD=\frac{1}{N_{p}}\sum |\frac{V_{orginal}-V_{additional}}{V_{orginal}}|$) of samples #6–7 compared with sample #5. Table 2 lists the *AARD*s of temperature responses, absorption/desorption volumes, pore-size distributions, and porosities. The detailed procedures of calculating the pore-size distribution and porosity will be introduced in the next section. Given the slight differences in the compositions of the samples, the results of the additional two cases (samples #6–7) echo relatively well with the base one (sample #5).

**Table 2:** The calculated AARDs of temperature, adsorption/desorption volume, pore-size distribution, and porosity between sample #5 and samples #6–7.

Sample	*AARD*s (%)				
	Temperature	Absorption volume	Desorption volume	Pore-size distribution	Porosity
Sample #6	4.4	3.58	16.50	16.2	6.5
Sample #7	4.5	17.9	5.63	12.4	10.4

## 4. Discussion

### 4.1 Evolution of pore structure under electromagnetic radiation

By using the obtained isothermal data, we use the Brunauer-Emmett-Teller (BET) method to calculate the total pore volume and specific surface area of the shale sample at different radiation times, as shown in Table 3. Results show that electromagnetic radiation significantly enhances the total pore volume and surface area because of the newly generated pores. The pore volume increases about four times after 5 min of electromagnetic radiation, and the specific area enhances about five times. Such a significant enhancement in pores changes the pattern of isotherms from H4 type to H1 type; similar observations have also been found in Wang et al. [19]. The increment in pore at early radiation times (before 1 min) is contributed by the evaporation of bound water [20]. Then, the organic matter is converted and decomposed to volatile contents, which promotes a significant number of new organic pores in the marine shale [21]. Also, micro-fractures in the matrix have been generated due to the induced thermal stress by electromagnetic radiation [22]. The mechanisms mentioned above contributed to the enhancement of total pore volume and specific surface area, which increase from 5.712×10^−3^ to 22.054×10^−3^ cm^3^/g, and 0.979 to and 5.123 m^2^/g, respectively.

**Table 3:** Pore volume, specific surface area, and types of isotherms of marine shale samples obtained at different electromagnetic radiation time.

Heating time, min	Total pore volume, 10^−3^ cm^3^/g	Specific surface area m^2^/g	Type of isotherm
0	5.712	0.979	H_4_
1	7.052	1.209	H_4_
2	8.815	1.484	H_4_
3	11.348	2.472	H_4_
4	14.703	3.415	H_1_
5	22.054	5.123	H_1_

**Fig 7:**
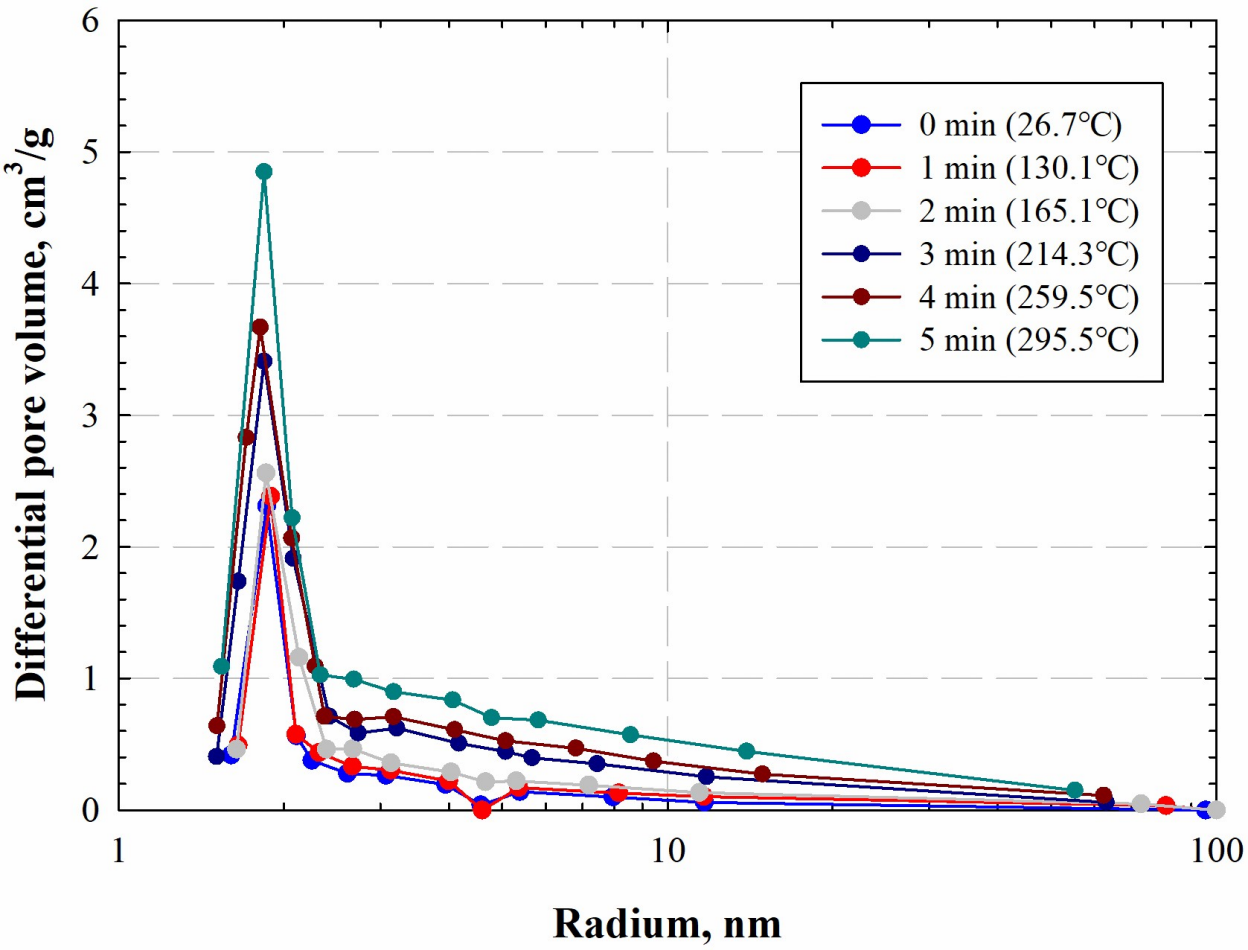
Pore-size distributions of marine shale samples obtained at different electromagnetic radiation times.

Moreover, we calculate the pore-size distributions of the marine shale sample by the Barrett-Joyner-Halenda method, which assumes the capillary condensation phenomenon in a cylindrical pore. Fig 7 shows the calculated pore-size distributions at different radiation times. Results show that the micropores and mesopores significantly enhance after electromagnetic radiation, suggesting that electromagnetic radiation can enlarge and generate organic pore. Similar phenomena have been observed in the research of Bai et al. [23].

### 4.2 Variation in porosity at different electromagnetic radiation time

**Fig 8:**
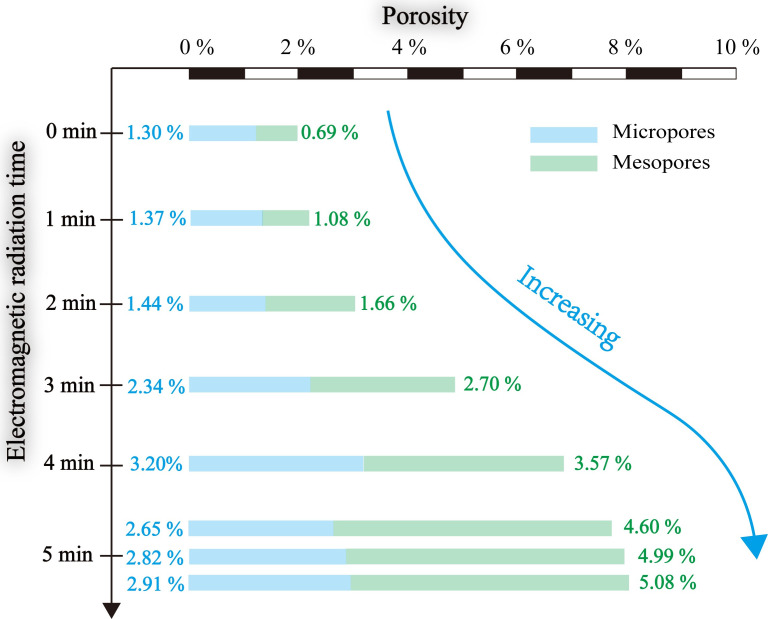
The variation of porosity of marine shale samples at different electromagnetic radiation times.

Porosity is a critical petrophysical property for the exploitation of shale resources. The electromagnetic radiation can significantly enhance the porosity of shale by dehydration of clay-bound water, decomposition of organic matter, and enlargement of pores by thermal expansion [14, 22]. By using the obtained pore-size distribution, we calculate the variations of porosity under electromagnetic radiation and specify the contribution of micropores and mesopores, as shown in Fig 8. After 5 min of electromagnetic radiation, the porosity sufficiently increases by about 5.84%; the micropores add about 4.3%, while the mesopores boost about 1.54%. The micropores continue to enhance in the first four minutes, which corresponding to the generation and enlargement of organic pores. However, the temperature gradually rises to high temperature as the radiation lasts to five minutes; consequently, the kerogen is decomposed to bitumen that is blocked the part of the pores, leading to reduced micropores. The mesopores steadily increase, which is caused by the vaporization of clay-bound water at the early stages and thermal expansion at the late stage.

### 4.3 Changes of pore morphology

In this section, the FE-SEM imaging analysis is performed to investigate the changes in the microstructure of shale samples under electromagnetic radiation experiments. By comparing the FE-SEM images of the raw sample and the ones that are exposed to electromagnetic radiation, we identified intensive newly developed organic pores and micro-fractures.

**Fig 9:**
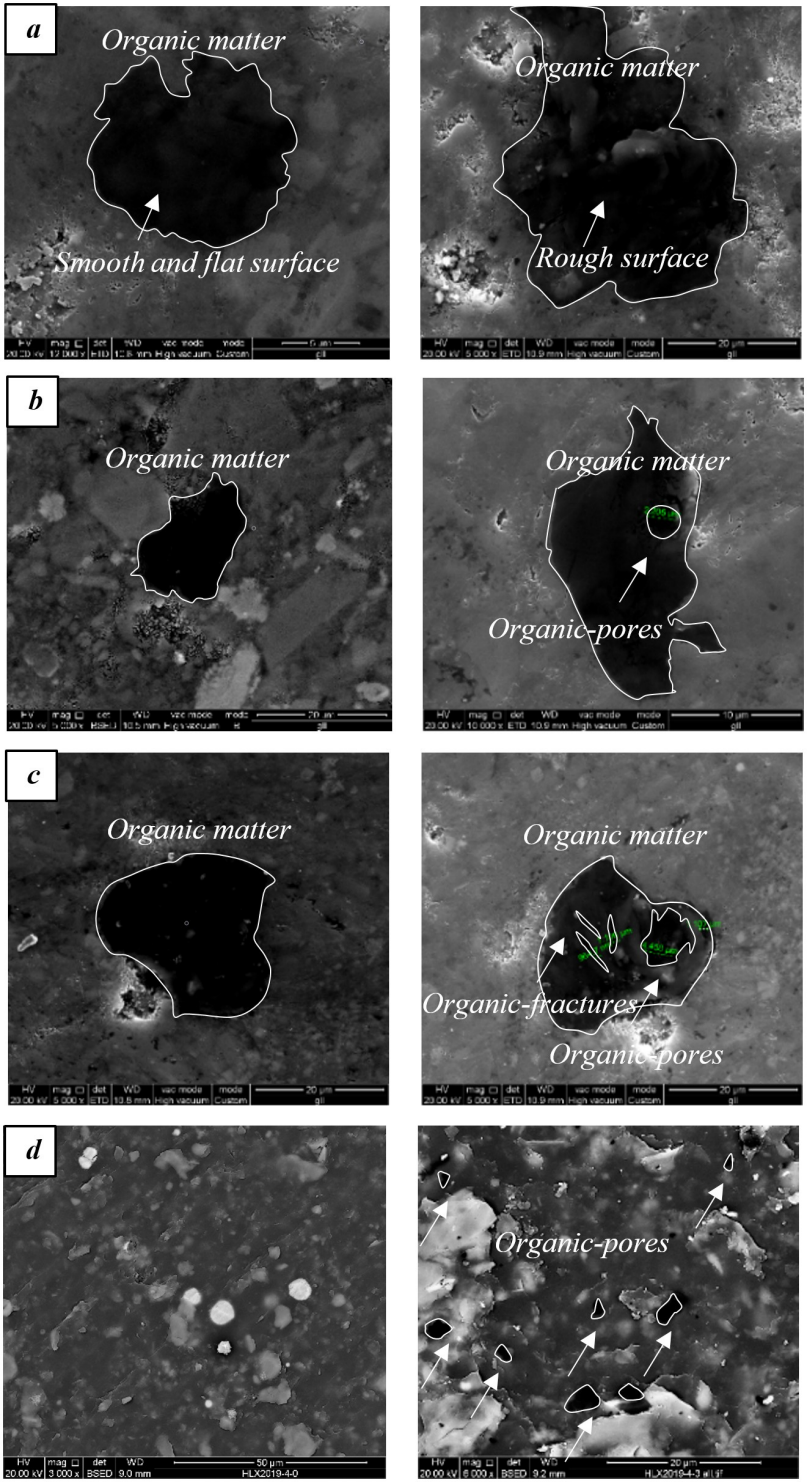
The SEM images obtained before and after 5 min of electromagnetic radiation. In these figures, the organic pores/fractures are highlighted in circles.

Fig 9 summarizes the FE-SEM images of shale samples before and after 5min of electromagnetic radiation, which focuses on the organic pores/fractures. Before the electromagnetic radiation, the morphology of pores is smooth and flat. However, after 5 min of electromagnetic radiation, the organic matter becomes rough and uneven. Also, new pores are generated within the organic matter, which tends to exhibit in a circular shape.

**Fig 10:**
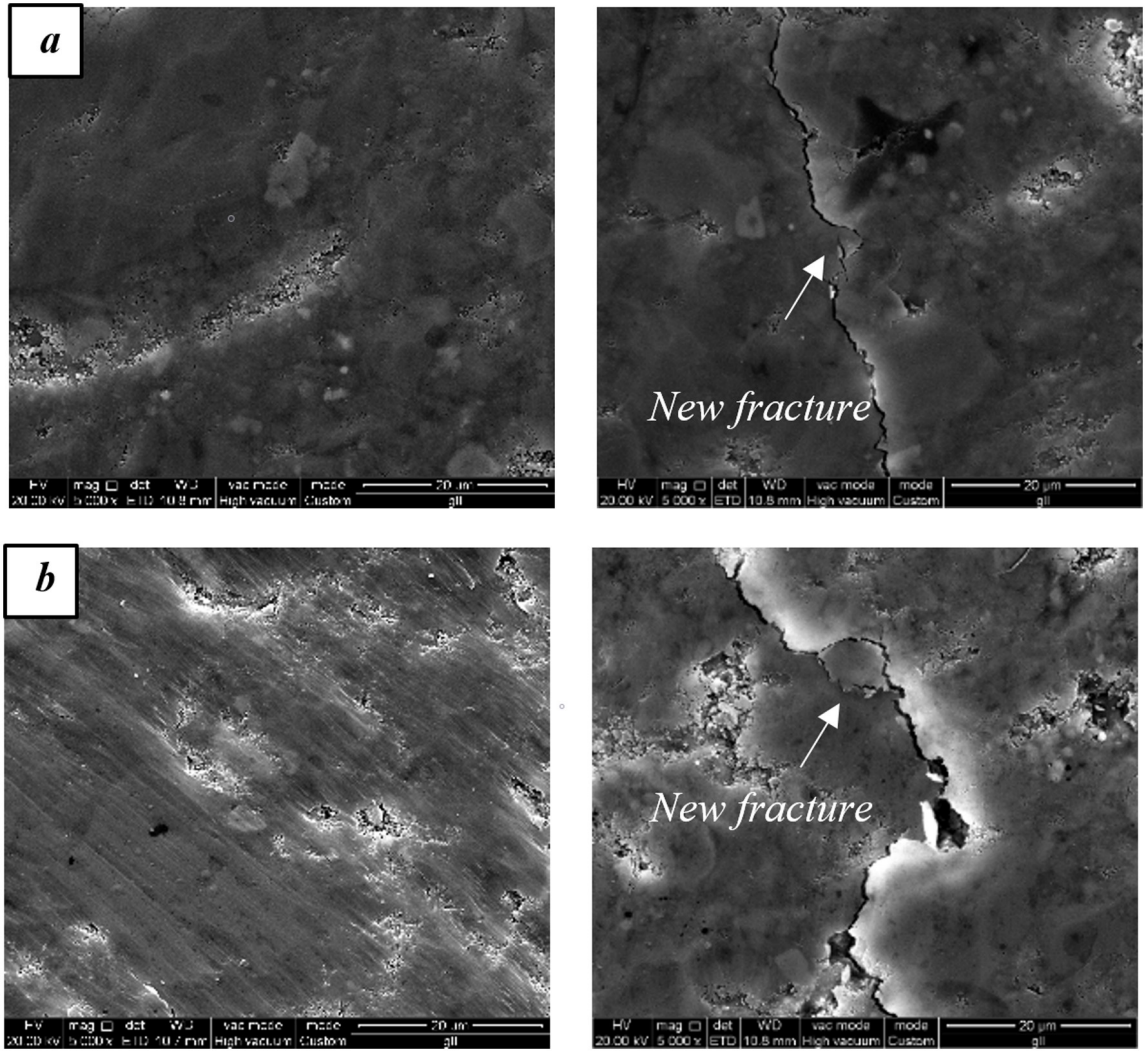
The SEM images showing the morphology and generated micro-fractures obtained before and after 5 min of electromagnetic radiation.

Fig 10 compares the morphology of clay minerals before and after electromagnetic radiation. The additional micro-fractures are observed in the clay-rich area of the sample. The clays are excellent electromagnetic energy absorber, whose thermal expansion induces stress and generates fractures [14].

### 4.4 Fractal dimension characterization

The evolution of pore structures of marine shale under electromagnetic radiation is a complex process, which involves multiphysics and several processes. To quantitively determine the pore structure changes, we performed fractal analyses to characterize the geometric and surface irregularity of marine shales in this process [24]. Fractal dimension is generally in the range of two to three; the minimum value represents smooth and homogeneous pores, while the maximum value indicates rough pore surface or heterogeneous pore structure [25]. For the fractal analyses of tight formation rocks, fractal dimension *D*_1_ (*P*_0_/*P*: 0~0.5) and *D*_2_ (*P*_0_/*P*: 0.5~1.0) are used to evaluate the degree of roughness and heterogeneity of pores [26]. The fractal dimension *D*_1_ can indicate the pore roughness, while *D*_2_ can represent the complexity of the pore structure [27, 28].

In this study, we use the Frenkel-Halsey-Hill (FHH) method and obtained nitrogen adsorption/desorption isotherm data to calculate the fractal dimension of shale samples [21, 29]. The FHH model is defined as follows [21]:

$$LnV=(D-3)Ln[Ln(\frac{P_{0}}{P})]+C$$ (1)

where *P* is equilibrium pressure, *P*_0_ is the vapor saturation pressure, *V* is the adsorption volume at equilibrium pressure, *C* is constant, and *D* is the fractal dimension. (*D*-3) can be obtained by the slope of the curve of LnV versus Ln[Ln(*P*_0_/*P*)].

**Fig 11:**
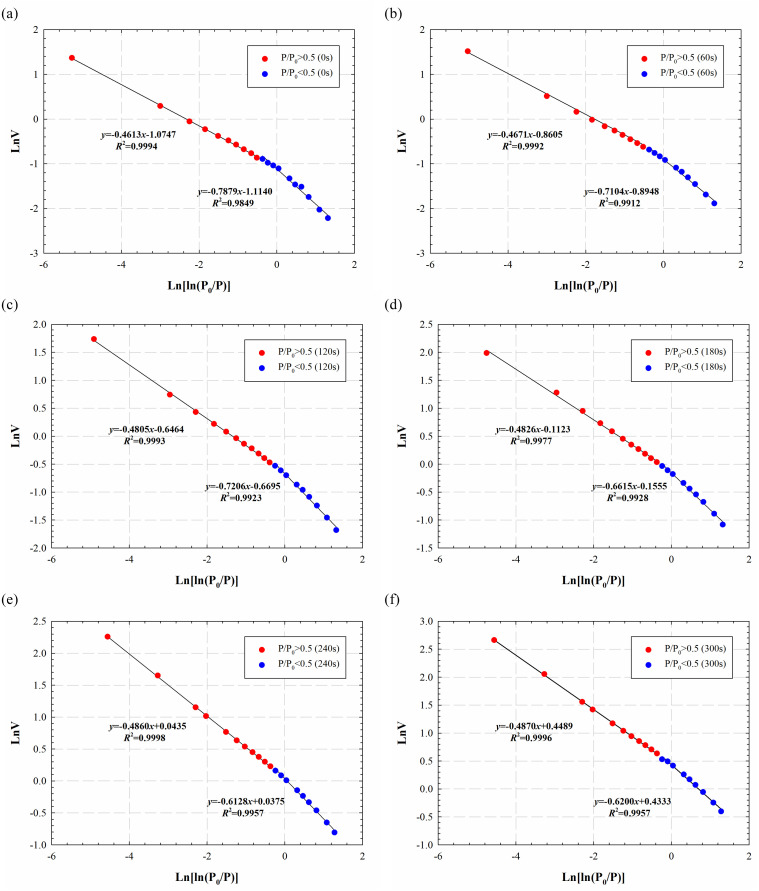
Relationships between Ln(*V*) and Ln(Ln(*P*_0_/*P*)) fitted by the FHH model for marine shale samples at different electromagnetic radiation times. In these figures, the blue ones are used to calculate the fractal dimension *D*_1_, while the red ones are applied to calculate the fractal dimension *D*_2_.

Fig 11 shows the relationships between Ln*V* and Ln [Ln (*P*_0_/*P*)] for the shale sample under different electromagnetic radiation times. The analyses are performed twice for the relative pressures smaller and larger than 0.5 to calculate *D*_1_ and *D*_2_, respectively. The obtained determination coefficients are higher than 0.98, showing a good fitting performance.

**Fig 12:**
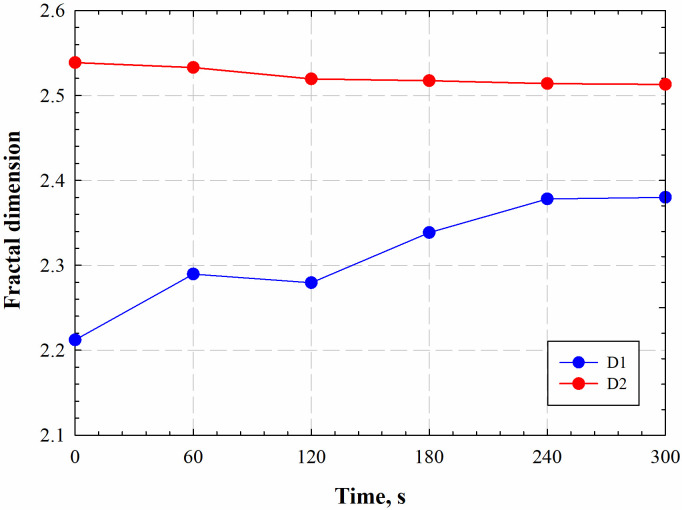
Variations in the fractal dimension of *D*_1_ and *D*_2_ for marine shale samples at different electromagnetic radiation time.

Fig 12 shows the variations of *D*_1_ and *D*_2_ at different radiation times. Results show that *D*_1_ steadily increases by electromagnetic radiation, while *D*_2_ slightly decreases under electromagnetic radiation. The increasing fractal dimension *D*_1_ indicates that the pores become rough after electromagnetic radiation, which echoes well the CT characterization by Tiwari et al. [30]. Meanwhile, the slightly decreased fractal dimension *D*_2_ suggests that the pore complexity of the shale sample is slightly reduced. Both experimental observations and fractal analyses reveal that electromagnetic radiation can significantly boost the pore space, demonstrating a great potential for enhancing shale oil and gas recovery.

## 5. Conclusions

In this study, a systematic study has been conducted to elucidate the pore structure changes of marine shale by electromagnetic radiation. The following conclusion can be drawn:

1. The shale samples exhibit a quick temperature rise under electromagnetic radiation; the surface temperature increases to about 300 °C after about 5 min of radiation. The temperature elevation provided by electromagnetic radiation causes dehydration of clay-bound water, thermal expansion, and decomposition of organic contents, which significantly enhances the pore spaces of marine shale samples.
2. The evolution of isothermal under electromagnetic radiation has been determined. Results show that the pore volume increases about four times after electromagnetic radiation, and the porosity enhances about 5.84%. The SEM characterization shows that organic pores are substantially generated by electromagnetic radiation, which significantly increases the micropores. However, part of the mesopores is blocked by the kerogen converted bitumen.
3. The fractal analyses have been performed to quantify the changes in the pore structure. Fractal dimensions *D*_1_ and *D*_2_ are calculated. The increasing *D*_1_ shows that the pores become rough after electromagnetic radiation.
4. The obtained experimental results and fractal analyses show a great potential of using electromagnetic radiation to enhance the pore spaces of marine shale, providing a non-aqueous stimulating technique for shale resource recovery.

## Supporting information

S1 Fig FE-SEM images. (DOCX)

S1 Table Temperature response data. (DOCX)

S2 Table N_2_ adsorption/desorption isotherms data. (DOCX)

S3 Table Fractal dimension data. (DOCX)

S4 Table The evolution of fractal dimensions under electromagnetic radiation. (DOCX)
