# Supplementary figures and images for: Evolution of pore structure and fractal characteristics of marine shale during electromagnetic radiation
Source: PLoS One. 2020 Oct 1;15(10):e0239662. doi: 10.1371/journal.pone.0239662 (PMC7529285; doi:10.1371/journal.pone.0239662)

**S1 Fig. FE-SEM images**


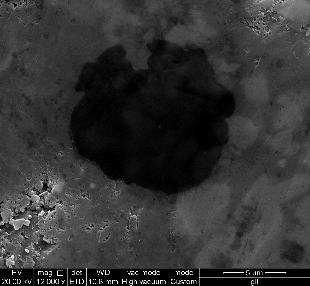

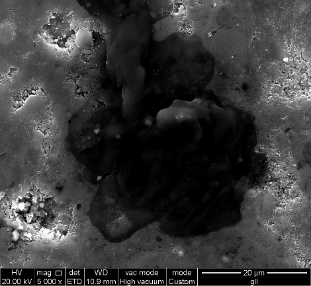


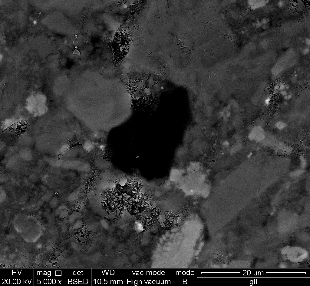

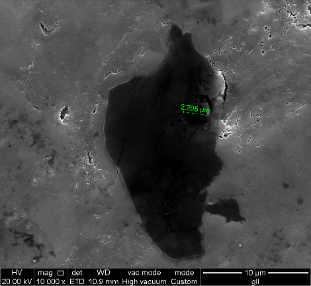


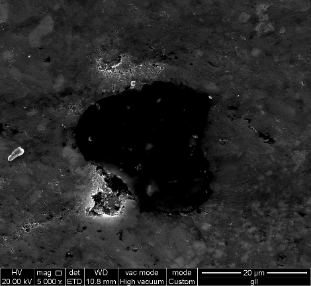

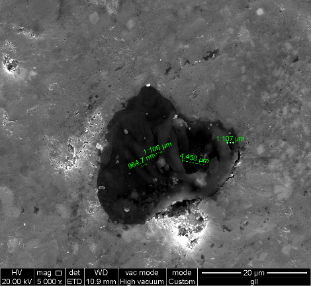


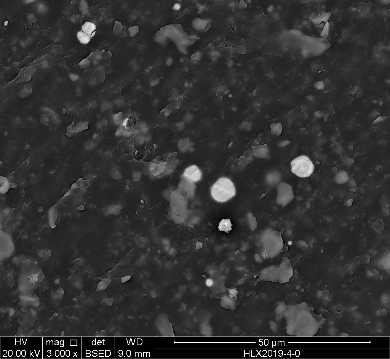

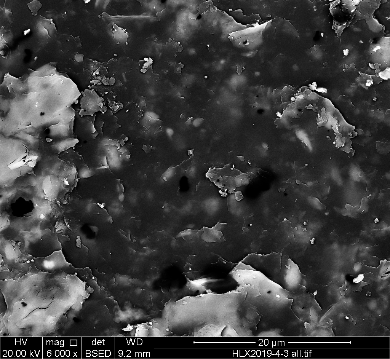


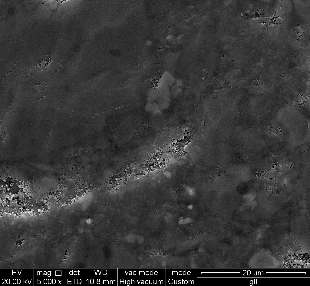

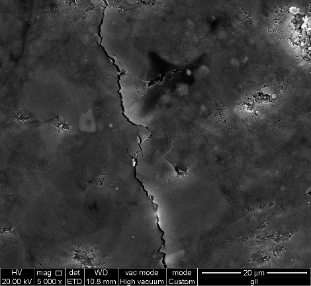


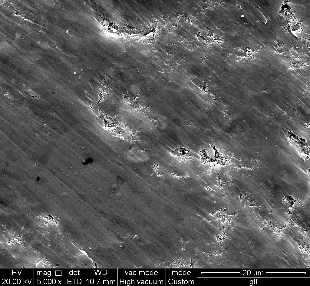

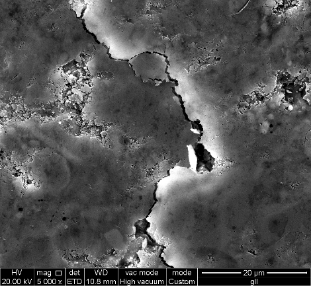

Supplement: S1 Fig — (DOCX) [file pone.0239662.s001.docx]
