# Supplementary material for: Evolution of pore structure and fractal characteristics of marine shale during electromagnetic radiation
Source: PLoS One. 2020 Oct 1;15(10):e0239662. doi: 10.1371/journal.pone.0239662 (PMC7529285; doi:10.1371/journal.pone.0239662)
Supplement: S1 Table — (DOCX) [file pone.0239662.s002.docx]

**S1 Table. Temperature response data**

| Heating time (min) | Temperature 1 (℃) | Temperature 2 (℃) | Temperature 3 (℃) | Averaged temperature  (℃) |
| --- | --- | --- | --- | --- |
| 0 | 23.7 | 25.8 | 30.6 | 26.7 |
| 1 | 130.1 | 123.1 | 137.0 | 130.1 |
| 2 | 167.1 | 152.2 | 176.2 | 165.1 |
| 3 | 210.1 | 205.6 | 228.6 | 214.1 |
| 4 | 259.5 | 254.1 | 265.1 | 259.5 |
| 5 | 290.5 | 287.0 | 309.6 | 295.5 |
