# Supplementary material for: Evolution of pore structure and fractal characteristics of marine shale during electromagnetic radiation
Source: PLoS One. 2020 Oct 1;15(10):e0239662. doi: 10.1371/journal.pone.0239662 (PMC7529285; doi:10.1371/journal.pone.0239662)
Supplement: S2 Table — (DOCX) [file pone.0239662.s003.docx]

**S2 Table. N_2_ adsorption/desorption isotherms data**

| Sample #0 (0 min) | | Sample #1 (1 min) | | Sample #2 (2 min) | | Sample #3 (3 min) | | Sample #4 (4 min) | | Sample #5 (5 min) | | Sample #6 (5 min) | | Sample #7 (5 min) | |
| --- | --- | --- | --- | --- | --- | --- | --- | --- | --- | --- | --- | --- | --- | --- | --- |
| Relative pressure | Volume  adsorbed | Relative pressure | Volume  adsorbed | Relative pressure | Volume  adsorbed | Relative pressure | Volume  adsorbed | Relative pressure | Volume  adsorbed | Relative pressure | Volume  adsorbed | Relative pressure | Volume  adsorbed | Relative pressure | Volume  adsorbed |
| 0.0240 | 0.1090 | 0.0245 | 0.1515 | 0.0233 | 0.1863 | 0.0235 | 0.3391 | 0.0274 | 0.4461 | 0.0274 | 0.6691 | 0.0258 | 0.6216 | 0.0258 | 0.4378 |
| 0.0500 | 0.1317 | 0.0508 | 0.1847 | 0.0518 | 0.2329 | 0.0494 | 0.4118 | 0.0524 | 0.5217 | 0.0524 | 0.7826 | 0.0536 | 0.7626 | 0.0536 | 0.5788 |
| 0.1030 | 0.1750 | 0.1044 | 0.2334 | 0.1015 | 0.2887 | 0.1023 | 0.5087 | 0.1044 | 0.6306 | 0.1044 | 0.9459 | 0.1039 | 0.9457 | 0.1038 | 0.8043 |
| 0.1520 | 0.2200 | 0.1524 | 0.2714 | 0.1534 | 0.3376 | 0.1512 | 0.5809 | 0.1557 | 0.7165 | 0.1557 | 1.0747 | 0.1534 | 1.0722 | 0.1534 | 0.8602 |
| 0.2010 | 0.2310 | 0.2011 | 0.3071 | 0.2047 | 0.3827 | 0.2023 | 0.6446 | 0.2028 | 0.7910 | 0.2028 | 1.1865 | 0.2030 | 1.1846 | 0.2057 | 1.0008 |
| 0.2500 | 0.2650 | 0.2502 | 0.3369 | 0.2568 | 0.4202 | 0.2550 | 0.7131 | 0.2523 | 0.8645 | 0.2523 | 1.2968 | 0.2512 | 1.2404 | 0.2572 | 1.0849 |
| 0.3510 | 0.3310 | 0.3517 | 0.4001 | 0.3544 | 0.4964 | 0.3543 | 0.8368 | 0.3526 | 1.0106 | 0.3526 | 1.5159 | 0.3394 | 1.4089 | 0.3232 | 1.1688 |
| 0.4020 | 0.3540 | 0.4014 | 0.4337 | 0.4059 | 0.5418 | 0.4034 | 0.8950 | 0.4025 | 1.0899 | 0.4025 | 1.6348 | 0.3992 | 1.5636 | 0.3992 | 1.3515 |
| 0.4520 | 0.3760 | 0.4504 | 0.4688 | 0.4588 | 0.5883 | 0.4541 | 0.9658 | 0.4554 | 1.1753 | 0.4554 | 1.7630 | 0.4543 | 1.6618 | 0.4562 | 1.4073 |
| 0.5000 | 0.4090 | 0.4998 | 0.5050 | 0.5078 | 0.6246 | 0.5026 | 1.0359 | 0.5013 | 1.2567 | 0.5013 | 1.8851 | 0.4976 | 1.7884 | 0.5010 | 1.4633 |
| 0.5500 | 0.4210 | 0.5520 | 0.5381 | 0.5541 | 0.6753 | 0.5531 | 1.1101 | 0.5495 | 1.3510 | 0.5495 | 2.0265 | 0.5451 | 1.8867 | 0.5492 | 1.5191 |
| 0.5960 | 0.4650 | 0.6003 | 0.5838 | 0.6008 | 0.7325 | 0.6017 | 1.2019 | 0.5992 | 1.4567 | 0.5992 | 2.1851 | 0.6008 | 2.0839 | 0.6055 | 1.7444 |
| 0.6520 | 0.5090 | 0.6521 | 0.6361 | 0.6522 | 0.8032 | 0.6513 | 1.3074 | 0.6464 | 1.5701 | 0.6464 | 2.3552 | 0.6449 | 2.2528 | 0.6483 | 1.9700 |
| 0.7040 | 0.5650 | 0.7050 | 0.7015 | 0.7043 | 0.8730 | 0.6999 | 1.4167 | 0.6993 | 1.7123 | 0.6993 | 2.5685 | 0.6985 | 2.4924 | 0.7006 | 2.1955 |
| 0.7500 | 0.6200 | 0.7523 | 0.7728 | 0.7509 | 0.9648 | 0.7505 | 1.5738 | 0.7477 | 1.8879 | 0.7477 | 2.8318 | 0.7488 | 2.7603 | 0.7624 | 2.5764 |
| 0.8020 | 0.6860 | 0.8018 | 0.8512 | 0.8013 | 1.0850 | 0.8051 | 1.7996 | 0.8022 | 2.1549 | 0.8022 | 3.2324 | 0.8031 | 3.1554 | 0.8405 | 3.2397 |
| 0.8540 | 0.7960 | 0.8528 | 0.9840 | 0.8507 | 1.2465 | 0.8516 | 2.0805 | 0.8767 | 2.7623 | 0.8767 | 4.1435 | 0.8771 | 4.0875 | 0.9029 | 4.0305 |
| 0.8999 | 0.9490 | 0.8990 | 1.1759 | 0.9040 | 1.5401 | 0.9030 | 2.5900 | 0.9042 | 3.1681 | 0.9042 | 4.7522 | 0.9085 | 4.7515 | 0.9382 | 4.7369 |
| 0.9515 | 1.3400 | 0.9515 | 1.6696 | 0.9494 | 2.1021 | 0.9493 | 3.5963 | 0.9627 | 5.2103 | 0.9627 | 7.8155 | 0.9694 | 7.7336 | 0.9654 | 6.4330 |
| 0.9949 | 3.9300 | 0.9935 | 4.5588 | 0.9927 | 5.6863 | 0.9914 | 7.3027 | 0.9896 | 9.5707 | 0.9896 | 14.356 | 0.9904 | 15.6253 | 0.9952 | 12.3846 |
| 0.9220 | 1.4100 | 0.9218 | 1.7606 | 0.9291 | 2.3267 | 0.9228 | 3.7063 | 0.9421 | 4.9389 | 0.9421 | 7.4084 | 0.9341 | 8.1966 | 0.5788 | 6.9989 |
| 0.8970 | 1.2990 | 0.8980 | 1.5998 | 0.8760 | 1.8957 | 0.8820 | 3.1151 | 0.8999 | 3.7783 | 0.8999 | 5.6674 | 0.8972 | 6.3155 | 0.8043 | 5.2748 |
| 0.8270 | 1.0580 | 0.8267 | 1.3203 | 0.8219 | 1.6487 | 0.8241 | 2.6053 | 0.8425 | 3.0321 | 0.8425 | 4.5482 | 0.8384 | 5.1655 | 0.8602 | 4.2435 |
| 0.7660 | 0.9700 | 0.7689 | 1.1875 | 0.7724 | 1.5008 | 0.7996 | 2.4601 | 0.7844 | 2.6011 | 0.7844 | 3.9016 | 0.7841 | 4.4355 | 1.0008 | 3.6787 |
| 0.6880 | 0.8490 | 0.7683 | 1.1933 | 0.7712 | 1.5026 | 0.7800 | 2.3559 | 0.7708 | 2.5273 | 0.7708 | 3.7910 | 0.7634 | 4.2492 | 1.0849 | 3.4388 |
| 0.6310 | 0.7710 | 0.6882 | 1.0583 | 0.6893 | 1.3173 | 0.6883 | 1.9863 | 0.6952 | 2.1777 | 0.6952 | 3.2666 | 0.6945 | 3.7682 | 1.1688 | 3.0721 |
| 0.5800 | 0.7490 | 0.6332 | 0.9786 | 0.6328 | 1.2112 | 0.6308 | 1.8114 | 0.6339 | 1.9736 | 0.6339 | 2.9604 | 0.6306 | 3.4115 | 1.3515 | 2.7337 |
| 0.5200 | 0.6800 | 0.5792 | 0.9127 | 0.5834 | 1.1273 | 0.6031 | 1.7517 | 0.5833 | 1.8318 | 0.5833 | 2.7477 | 0.5811 | 3.1479 | 1.4073 | 2.5789 |
| 0.4980 | 0.6500 | 0.5296 | 0.8523 | 0.5508 | 1.0928 | 0.5360 | 1.5963 | 0.5317 | 1.7077 | 0.5317 | 2.4616 | 0.5295 | 2.8532 | 1.4633 | 2.3676 |
| 0.4360 | 0.4850 | 0.5019 | 0.8163 | 0.4896 | 0.9456 | 0.4852 | 1.3861 | 0.4865 | 1.5571 | 0.4865 | 2.2357 | 0.4833 | 2.5895 | 1.5191 | 2.1561 |
| 0.3840 | 0.4190 | 0.4386 | 0.5915 | 0.4343 | 0.7339 | 0.4358 | 1.1272 | 0.4345 | 1.2888 | 0.4345 | 1.9332 | 0.4405 | 2.3725 | 1.7444 | 1.9165 |
| 0.3490 | 0.4090 | 0.3815 | 0.5284 | 0.3833 | 0.6716 | 0.3850 | 1.0304 | 0.3837 | 1.1869 | 0.3837 | 1.7804 | 0.3869 | 2.1867 | 1.9700 | 1.7901 |
| 0.2800 | 0.3630 | 0.3490 | 0.5034 | 0.3358 | 0.6211 | 0.3547 | 0.9849 | 0.3495 | 1.1319 | 0.3495 | 1.6979 | 0.3503 | 2.0318 | 2.1955 | 1.7057 |
| 0.2200 | 0.3190 | 0.2827 | 0.4490 | 0.2813 | 0.5658 | 0.2875 | 0.8929 | 0.3048 | 1.0622 | 0.3048 | 1.5933 | 0.3041 | 1.8769 | 2.5764 | 1.5932 |
| 0.1740 | 0.2800 | 0.2276 | 0.4012 | 0.2517 | 0.5423 | 0.2340 | 0.8135 | 0.2375 | 0.9595 | 0.2375 | 1.4393 | 0.2371 | 1.7691 | 3.2397 | 1.4669 |
| 0.1270 | 0.2410 | 0.1759 | 0.3571 | 0.1810 | 0.4734 | 0.1842 | 0.7403 | 0.1805 | 0.8675 | 0.1805 | 1.3013 | 0.1805 | 1.5523 | 4.0305 | 1.3123 |
| 0.0780 | 0.2190 | 0.1290 | 0.3154 | 0.1346 | 0.4204 | 0.1333 | 0.6523 | 0.1335 | 0.7871 | 0.1335 | 1.1806 | 0.1344 | 1.4441 | 4.7369 | 1.1434 |
| 0.0490 | 0.1640 | 0.0793 | 0.2623 | 0.0799 | 0.3557 | 0.0800 | 0.5536 | 0.0811 | 0.6857 | 0.0811 | 1.0286 | 0.0801 | 1.2584 | 6.4330 | 0.9602 |
| 0.0200 | 0.1310 | 0.0443 | 0.2141 | 0.0460 | 0.3007 | 0.0450 | 0.4633 | 0.0460 | 0.5966 | 0.0460 | 0.8949 | 0.0441 | 1.1812 | 12.3846 | 0.8900 |
|  |  | 0.0284 | 0.1859 | 0.0286 | 0.2626 | 0.0284 | 0.4039 | 0.0296 | 0.5368 | 0.0296 | 0.8052 | 0.0244 | 1.0260 | 0.0238 | 0.7630 |
