# Supplementary material for: Evolution of pore structure and fractal characteristics of marine shale during electromagnetic radiation
Source: PLoS One. 2020 Oct 1;15(10):e0239662. doi: 10.1371/journal.pone.0239662 (PMC7529285; doi:10.1371/journal.pone.0239662)
Supplement: S3 Table — (DOCX) [file pone.0239662.s004.docx]

**S3 Table. Fractal dimension data**

| Sample #0, 0 min | | | Sample #1, 1 min | | | Sample #2, 2 min | | |
| --- | --- | --- | --- | --- | --- | --- | --- | --- |
| P/P0 | Ln[ln(P0/P)] | Ln V | P/P0 | Ln[ln(P0/P)] | Ln V | P/P0 | Ln[ln(P0/P)] | Ln V |
| 0.024 | 1.31633 | -2.21641 | 0.025 | 1.31076 | -1.88717 | 0.023 | 1.32465 | -1.68040 |
| 0.050 | 1.09719 | -2.02723 | 0.051 | 1.09186 | -1.68902 | 0.052 | 1.08553 | -1.45715 |
| 0.103 | 0.82111 | -1.74297 | 0.104 | 0.81524 | -1.45500 | 0.102 | 0.82749 | -1.24237 |
| 0.152 | 0.63333 | -1.51413 | 0.152 | 0.63183 | -1.30416 | 0.153 | 0.62840 | -1.08589 |
| 0.201 | 0.47278 | -1.46534 | 0.201 | 0.47262 | -1.18058 | 0.205 | 0.46149 | -0.96050 |
| 0.250 | 0.32663 | -1.32803 | 0.250 | 0.32615 | -1.08797 | 0.257 | 0.30697 | -0.86702 |
| 0.351 | 0.04590 | -1.10564 | 0.352 | 0.04393 | -0.91604 | 0.354 | 0.03659 | -0.70037 |
| 0.402 | -0.09288 | -1.03846 | 0.401 | -0.09114 | -0.83540 | 0.406 | -0.10349 | -0.61286 |
| 0.452 | -0.23058 | -0.97817 | 0.450 | -0.22619 | -0.75758 | 0.459 | -0.24955 | -0.53052 |
| 0.500 | -0.36651 | -0.89404 | 0.500 | -0.36592 | -0.68320 | 0.508 | -0.38906 | -0.47064 |
| 0.550 | -0.51444 | -0.86512 | 0.552 | -0.52045 | -0.61971 | 0.554 | -0.52706 | -0.39260 |
| 0.596 | -0.65872 | -0.76572 | 0.600 | -0.67279 | -0.53820 | 0.601 | -0.67423 | -0.31129 |
| 0.652 | -0.84931 | -0.67531 | 0.652 | -0.84968 | -0.45240 | 0.652 | -0.84993 | -0.21915 |
| 0.704 | -1.04703 | -0.57093 | 0.705 | -1.05090 | -0.35453 | 0.704 | -1.04818 | -0.13582 |
| 0.750 | -1.24590 | -0.47804 | 0.752 | -1.25662 | -0.25773 | 0.751 | -1.25009 | -0.03583 |
| 0.802 | -1.51119 | -0.37688 | 0.802 | -1.51033 | -0.16111 | 0.801 | -1.50703 | 0.08158 |
| 0.854 | -1.84627 | -0.22816 | 0.853 | -1.83742 | -0.01613 | 0.851 | -1.82178 | 0.22034 |
| 0.900 | -2.24931 | -0.05235 | 0.899 | -2.23959 | 0.16203 | 0.904 | -2.29293 | 0.43185 |
| 0.952 | -3.00144 | 0.29267 | 0.951 | -3.00112 | 0.51258 | 0.949 | -2.95737 | 0.74294 |
| 0.995 | -5.27596 | 1.36864 | 0.994 | -5.03672 | 1.51706 | 0.993 | -4.91169 | 1.73806 |

**S3 Table. Fractal dimension data (continued)**

| Sample #3, 3 min | | | Sample #4, 4 min | | | Sample #5, 5 min | | |
| --- | --- | --- | --- | --- | --- | --- | --- | --- |
| P/P0 | Ln[ln(P0/P)] | Ln V | P/P0 | Ln[ln(P0/P)] | Ln V | P/P0 | Ln[ln(P0/P)] | Ln V |
| 0.023 | 1.32233 | -1.08146 | 0.027 | 1.28061 | -0.80729 | 0.027 | 1.28061 | -0.40182 |
| 0.049 | 1.10108 | -0.88722 | 0.052 | 1.08113 | -0.65060 | 0.052 | 1.08113 | -0.24513 |
| 0.102 | 0.82417 | -0.67590 | 0.104 | 0.81500 | -0.46108 | 0.104 | 0.81500 | -0.05562 |
| 0.151 | 0.63603 | -0.54318 | 0.156 | 0.62049 | -0.33342 | 0.156 | 0.62049 | 0.07204 |
| 0.202 | 0.46890 | -0.43913 | 0.203 | 0.46721 | -0.23446 | 0.203 | 0.46721 | 0.17101 |
| 0.255 | 0.31225 | -0.33813 | 0.252 | 0.32013 | -0.14557 | 0.252 | 0.32013 | 0.25990 |
| 0.354 | 0.03701 | -0.17817 | 0.353 | 0.04166 | 0.01054 | 0.353 | 0.04166 | 0.41601 |
| 0.403 | -0.09679 | -0.11093 | 0.403 | -0.09435 | 0.08606 | 0.403 | -0.09435 | 0.49152 |
| 0.454 | -0.23634 | -0.03480 | 0.455 | -0.24004 | 0.16155 | 0.455 | -0.24004 | 0.53063 |
| 0.503 | -0.37390 | 0.03527 | 0.501 | -0.37015 | 0.22852 | 0.501 | -0.37015 | 0.63398 |
| 0.553 | -0.52384 | 0.10445 | 0.550 | -0.51304 | 0.30085 | 0.550 | -0.51304 | 0.70631 |
| 0.602 | -0.67723 | 0.18390 | 0.599 | -0.66923 | 0.37620 | 0.599 | -0.66923 | 0.78166 |
| 0.651 | -0.84677 | 0.26804 | 0.646 | -0.82924 | 0.45116 | 0.646 | -0.82924 | 0.85663 |
| 0.700 | -1.03066 | 0.34833 | 0.699 | -1.02815 | 0.53786 | 0.699 | -1.02815 | 0.94332 |
| 0.751 | -1.24845 | 0.45349 | 0.748 | -1.23521 | 0.63545 | 0.748 | -1.23521 | 1.04091 |
| 0.805 | -1.52869 | 0.58756 | 0.802 | -1.51206 | 0.76776 | 0.802 | -1.51206 | 1.17322 |
| 0.852 | -1.82835 | 0.73261 | 0.877 | -2.02839 | 1.01608 | 0.877 | -2.02839 | 1.42154 |
| 0.903 | -2.28258 | 0.95166 | 0.904 | -2.29550 | 1.15314 | 0.904 | -2.29550 | 1.55861 |
| 0.949 | -2.95530 | 1.27991 | 0.963 | -3.26971 | 1.65064 | 0.963 | -3.26971 | 2.05611 |
| 0.991 | -4.75484 | 1.98824 | 0.990 | -4.55976 | 2.25870 | 0.990 | -4.55976 | 2.66417 |
