# Supplementary material for: Evolution of pore structure and fractal characteristics of marine shale during electromagnetic radiation
Source: PLoS One. 2020 Oct 1;15(10):e0239662. doi: 10.1371/journal.pone.0239662 (PMC7529285; doi:10.1371/journal.pone.0239662)
Supplement: S4 Table — (DOCX) [file pone.0239662.s005.docx]

**S4 Table. The evolution of fractal dimensions under electromagnetic radiation**

| Heating time (min) | D1 | D2 |
| --- | --- | --- |
| 0 | 2.2121 | 2.5387 |
| 1 | 2.2896 | 2.5329 |
| 2 | 2.2794 | 2.5195 |
| 3 | 2.3385 | 2.5174 |
| 4 | 2.3782 | 2.5140 |
| 5 | 2.3800 | 2.5130 |
